# Medical staffing: Critical shortages on the horizon

**DOI:** 10.4103/2152-7806.63899

**Published:** 2010-05-31

**Authors:** Edward E Gordon

**Affiliations:** Imperial Consulting Corporation, Chicago, IL, USA

## GUEST EDITORIAL ON SOCIOECONOMICS

*Surgical Neurology International* will invite guest experts in different fields to provide brief information for all our readers worldwide.

Edward E. Gordon is an internationally recognized writer, researcher, speaker and consultant on jobs and the future of the United States and global workforce. He is the author of *Winning the Global Talent Showdown: How Businesses and Communities Can Partner to Rebuild the Jobs Pipeline *(Berrett-Koehler Publishers, 2009), and president of Imperial Consulting Corporation.

## MEDICAL STAFFING: CRITICAL SHORTAGES ON THE HORIZON

Between 2010 and 2020, nations all over the globe will experience profound changes in employment because of scientific and technological advances. Technology shows no signs of slowing down. Breathtaking developments seem just over the horizon. Nanotechnology is producing innovations on a molecular scale. Robots are proliferating in many settings.

Though globalization has meant increased mobility for businesses, service organizations and workers, the regrettable truth is that a finite pool of talent exists worldwide, and it falls significantly short of meeting worldwide demands. This is particularly true in STEM (science, technology, engineering or mathematical) areas. At the top of this STEM jobs pyramid are scientists, physicians and engineers with advanced degrees who are inventing new technologies and treatments. However, installing, applying and maintaining these technologies require an even broader base of middle jobs for knowledge technologists. The reasons for these shortages differ among nations; but in many developed counties, including the United States, the shortage stems from antiquated 20^th^-century education-to-employment systems being out-of-sync with the employment requirements of the 21^st^-century high-tech economies.

The rapid growth of the retirement-age populations in many developed countries will put increasing strains on health care systems. In the United States, the recession provided some temporary relief in talent demand in the health care sector. It both delayed some baby-boomer retirements and curtailed elective procedures. However, between 2010 and 2020, a tsunami of technicians, pharmacists, nurses, therapists and physicians will retire, further straining already inadequate health care staffing in many areas. The recent passage of “Obama Care” health legislation will exacerbate these talent shortages, while thus far offering little systemic relief toward providing more educational programs and incentives for the preparation of medical professionals at all levels.

There are, however, around the world businesses, service organizations and communities that have begun working to change this talent black hole into a decade of opportunity. They are beginning to create community-based organizations (CBOs) that I call “Gateways-to-the Future.” CBOs are partnerships among a broad spectrum of community groups, including health care organizations, to rebuild the jobs pipeline by reinventing local education-to-employment systems, retraining workers, and providing information on in-demand careers and the education and training needed for them. CBOs are vitally important because the health care sector by itself cannot solve the problem of its chronic talent deficit. Action at the local and regional levels can be an effective method for effecting change from the ground up.

